# Checklist of the familes Chaoboridae, Dixidae, Thaumaleidae, Psychodidae and Ptychopteridae (Diptera) of Finland

**DOI:** 10.3897/zookeys.441.7532

**Published:** 2014-09-19

**Authors:** Jukka Salmela, Lauri Paasivirta, Gunnar M. Kvifte

**Affiliations:** 1 Metsähallitus, Natural Heritage Services, P.O. Box 8016, FI-96101 Rovaniemi, Finland; 2 Ruuhikoskenkatu 17 B 5, 24240 Salo, Finland; 3 Department of Limnology, University of Kassel, Heinrich-Plett-Str. 40, 34132 Kassel-Oberzwehren, Germany

**Keywords:** Finland, Diptera, species list, biodiversity, faunistics

## Abstract

A checklist of the families Chaoboridae, Dixidae, Thaumaleidae, Psychodidae and Ptychopteridae (Diptera) recorded from Finland is given. Four species, *Dixella dyari* Garret, 1924 (Dixidae), *Threticus tridactilis* (Kincaid, 1899), *Panimerus albifacies* (Tonnoir, 1919) and *Panimerus przhiboroi* Wagner, 2005 (Psychodidae) are reported for the first time from Finland.

## Introduction

Psychodidae or moth flies are an intermediately diverse family of nematocerous flies, comprising over 3000 species world-wide (Pape et al. 2011). Its taxonomy is still very unstable, and multiple conflicting classifications exist (Duckhouse 1987, Vaillant 1990, Ježek and van Harten 2005). The nomenclature used in the Finnish check-list follows the Norwegian one (Kvifte et al. 2011). For differences between the taxonomy used herein and that used in previous works on Finnish Psychodidae (e.g. Hackman 1980, Salmela 2003, 2005, Salmela et al. 2007, Autio and Salmela 2010) see Kvifte et al. (2011). Two species, *Pneumia nubila* and *Pneumia palustris*, listed in the previous check-lists by Hackman (1980) and Wagner (2013) have not been confirmed through study of material. Of these, *Pneumia palustris* have been suggested to be synonymous with *Pneumia gracilis* (Eaton, 1893) (Withers 1989). Both these species have been linked to some taxonomic confusion in the past, and thus the record of *Pneumia nubila* may represent *Pneumia trivialis*. The present check-list contains the first Finnish records of the species *Threticus tridactilis* (Kincaid, 1899), *Panimerus albifacies* (Tonnoir, 1919) and *Panimerus przhiboroi* Wagner, 2005 (see Notes).

Finnish Psychodidae are very well studied compared to other Nordic countries, however new species are still being discovered at a regular rate. Most of these are new country records, but in recent years the fauna has also yielded some species new to science (Salmela et al. 2012, Kvifte unpubl.). This trend is likely to continue. Probably between twelve and thirty further species can be expected to be found in the Finnish fauna. There are no good identification sources available for Psychodids, as all works suffer from taxonomic or nomenclatural errors and/or insufficient descriptions. The best works relevant to the Finnish fauna are Vaillant (1971-1983), Withers (1989) and Wagner (1997c, genera only).

Phantom midges (Chaoboridae) are close relatives to mosquitoes (Culicidae) and meniscus midges (Dixidae) (Saether 1970, 1997). There are around 50 known species of phantom midges in the world and 15 in the palearctic region, of which five are holoarctic species. In Finland, there are eight species in three genera (Hirvenoja 1960, Hackman 1980, in which the species in the genus *Mochlonyx* were still doubtful).

The only strictly lacustrine species in Finland is *Chaoborus flavicans*. The other species live in small fishless lakes, ponds and springs where their predators are mainly dragon flies, dytiscid beetles and frogs. *Chaoborus nyblaei* and *Cryophila lapponica* are found from small fishless ponds on fells and ponds created by melting water. However, in 2013 larvae of *Chaoborus nyblaei* were found from the boreal zone, NE Lapland (Savukoski) from a small but permanent pond. *Chaoborus pallidus* lives in eutrophic lakes and small lakes in southern and central Finland (Hirvenoja 1960, 1963, Paasivirta 2002). However, there have not been any systematic surveys of the fauna across the biogeographic provinces and the conservation statuses of the species have not been evaluated. Finnish species can be identified by using Saether (1997, 2002).

Meniscus midges (Dixidae) belong to infraorder Culicomorpha. There are nearly 200 known species of which 53 occur in the Palaearctic region. In Finland two genera and 16 species are present (Hackman 1980, Salmela 2003, Salmela et al. 2007). One species (*Dixella dyari* Garret, 1924) is reported here for the first time from Finland (see Notes). Larvae of meniscus midges dwell in or slightly above water meniscus, having a characteristic U-shaped position (Wagner 1997a). Members of the genus *Dixella* are associated with lentic or slow flowing waters, such as ponds, lake shores and bog pools. Species of the genus *Dixa* are lotic, dwelling in running waters of varying size. Two Finnish species, *Dixa dilatata* and *Dixa submaculata* prefer springs and spring brooks. The Finnish fauna is generally rather well known, although three species have hitherto been collected from single localities. One northern *Dixa* species close to *Dixa dilatata* is still undescribed. A majority of the species can be identified by using Martini (1928), Peus (1934, 1936), Vaillant (1967) and Disney (1999).

Trickle midges (Thaumaleidae) is a small family belonging to infraorder Culicomorpha. Globally, nearly 200 species are known of which 76 are European. Central European mountains harbor a rich assemblage of trickle midges (Wagner 2002), at least if compared to the poor diversity (5 spp.) in Fennoscandian area (Wagner 1997b, Andersen et al. 2013). Only one species, *Thaumalea truncata*, has been hitherto recorded from Finland (see the discussion in Andersen et al. 2013). Larvae of trickle midges dwell on hygropetric substrates or splash zones of running waters (Wagner 1997b). *Thaumalea truncata* occurs mainly in northern Finland, both boreal and subarctic ecoregions, and adult specimens have been caught around springs and headwater streams (Salmela 2003, Salmela 2008).

Phantom crane flies (Ptychopteridae) belong to infraorder Ptychopteromorpha, being a sister group of Psychdomorpha and Culicomorpha (Wiegmann et al. 2011). Ptychopteridae is a small family with over 69 species in the world and 14 in Europe. Seven species are known from Finland, all belonging to the genus *Ptychoptera*. Only one Finnish species, *Ptychoptera minuta*, is widespread, locally abundant and almost ubiquitous. Other species are much less frequent, occurring in peatlands, headwater streams, springs and swamps. Adult *Ptychoptera* superficially resemble Tipuloidea crane flies, being slender and having long legs. Larvae of *Ptychoptera* live in shallow aquatic habitats, usually in soft substrates (Andersson 1997). Finnish species can be identified by using Andersson (1997) and Krzeminski (1986).

**Table 1.** Number of species by family.

Family	Number of species in			Level of knowledge
	World (Pape et al. 2011)	Europe	Finland	
**Psychodomorpha:**				
Psychodidae	2958	493	61–63	average
**Culicomorpha:**				
Chaoboridae	54	9	8	good
Dixidae	186	32	16	average
Thaumaleidae	182	76	1	good
**Ptychopteromorpha:**				
Ptychopteridae	69 (Wagner et al. 2008)	14	7	good

## Checklist

Nematocera Dumeril, 1805

infraorder Psychodomorpha Hennig, 1968

**PSYCHODIDAE** Newman, 1834

PSYCHODINAE Newman, 1834

tribe Pericomaini Enderlein, 1935

***PARABAZARELLA*** Vaillant, 1983

*Parabazarella subneglecta* (Tonnoir, 1922)

***BERDENIELLA*** Vaillant, 1976

*Berdeniella freyi* (Berdén, 1954)

***CLYTOCERUS*** Eaton, 1904

**sg. *Boreoclytocerus*** Duckhouse, 1978

*Clytocerus ocellaris* (Meigen, 1818)

*Clytocerus rivosus* (Tonnoir, 1919)

*Clytocerus tetracorniculatus* Wagner, 1977

***PERICOMA*** Walker, 1856

**sg. *Pachypericoma*** Vaillant, 1978

*Pericoma blandula* Eaton, 1893

*Pericoma nielseni* Kvifte, 2010

= *formosa* Nielsen, 1964 (preocc. Meunier, 1905)

**sg. *Pericoma*** Walker, 1856

*Pericoma rivularis* Berdén, 1954

***PNEUMIA*** Enderlein, 1935

*Pneumia borealis* (Berdén, 1954)

*Pneumia bucegica* (Vaillant, 1981)

*Pneumia mutua* (Eaton, 1893)

? *Pneumia nubila* (Meigen, 1818)

? *Pneumia palustris* (Meigen, 1818)

*Pneumia pilularia* (Tonnoir, 1940)

*Pneumia stammeri* (Jung, 1954)

*Pneumia trivialis* (Eaton, 1893)

*Pneumia ussurica* (Wagner, 1994)

***ULOMYIA*** Walker, 1856

*Ulomyia cognata* (Eaton, 1893)

*Ulomyia fuliginosa* (Meigen, 1804)

tribe Psychodini Newman, 1834

***PHILOSEPEDON*** Eaton, 1904

*Philosepedon balkanicus* Krek, 1970

*Philosepedon humeralis* (Meigen, 1818)

*Philosepedon soljani* Krek, 1971

***PSYCHODA*** Latreille, 1796

*Psychoda albipennis* Zetterstedt, 1850

= *parthenogenetica* Tonnoir, 1940

*Psychoda alternata* Say, 1824

*Psychoda brevicornis* Tonnoir, 1940

*Psychoda buxtoni* Withers, 1988

*Psychoda cinerea* Banks, 1894

*Psychoda crassipenis* Tonnoir, 1940

*Psychoda cultella* Salmela, Kvifte & More, 2012

*Psychoda erminea* Eaton, 1898

*Psychoda gemina* (Eaton, 1904)

*Psychoda grisescens* Tonnoir, 1922

*Psychoda itoco* Tokunaga & Komyo, 1955

*Psychoda lativentris* Berdén, 1952

*Psychoda lobata* Tonnoir, 1940

*Psychoda minuta* Banks, 1894

*Psychoda phalaenoides* Linnaeus, 1758

*Psychoda satchelli* Quate, 1955

*Psychoda setigera* Tonnoir, 1922

*Psychoda trinodulosa* Tonnoir, 1922

*Psychoda uniformata* Haseman, 1907

***THRETICUS*** Eaton, 1904

*Threticus tridactilis* (Kincaid, 1899)

***TRICHOPSYCHODA*** Tonnoir, 1922

*Trichopsychoda hirtella* (Tonnoir, 1919)

tribe Maruinini Enderlein, 1937

***LOBULOSA*** Szabo, 1960

*Lobulosa pollex* (Berdén, 1954)

***TONNOIRIELLA*** Vaillant, 1971

*Tonnoiriella nigricauda* (Tonnoir, 1919)

tribe Mormiini Enderlein, 1937

***MORMIA* Enderlein, 1935**

*Mormia niesiolowskii* Wagner, 1985

*Mormia strobli* (Ježek, 1986)

tribe Paramormiini Enderlein, 1937

***PARAJUNGIELLA*** Vaillant, 1972

*Parajungiella consors* (Eaton, 1893)

*Parajungiella ellisi* (Withers, 1987)

*Parajungiella pseudolongicornis* Wagner, 1975

***LEPISEODINA*** Enderlein, 1937

*Lepiseodina rothschildi* (Eaton, 1912)

***PANIMERUS*** Eaton, 1913

**sg. *Panimerus*** Eaton, 1913

*Panimerus albomaculatus* (Wahlgren, 1904)

*Panimerus notabilis* (Eaton, 1893)

**sg. *Psycmera*** Ježek, 1984

*Panimerus albifacies* (Tonnoir, 1919)

*Panimerus intellegus* (Jung, 1956)

*Panimerus przhiboroi* Wagner, 2005

***PARAMORMIA*** Enderlein, 1935

*Paramormia polyscoidea* (Krek, 1970)

*Paramormia ustulata* (Walker, 1856)

***PERIPSYCHODA*** Enderlein, 1935

*Peripsychoda auriculata* (Curtis, 1839)

*Peripsychoda fusca* (Macquart, 1826)

***TELMATOSCOPUS*** Eaton, 1904

*Telmatoscopus advena* (Eaton, 1893)

*Telmatoscopus similis* Tonnoir, 1922

SYCORACINAE Rondani, 1856

***SYCORAX*** Haliday in Curtis, 1839

*Sycorax silacea* Haliday in Curtis, 1839

infraorder Culicomorpha Hennig, 1948

superfamily Culicoidea Meigen, 1818

**CHAOBORIDAE** Newman, 1834

***CHAOBORUS*** Lichtenstein, 1800

**sg. *Chaoborus*** Lichtenstein, 1800

*Chaoborus crystallinus* (De Geer, 1776)

*Chaoborus flavicans* (Meigen, 1830)

*Chaoborus obscuripes* (van der Wulp, 1859)

**sg. *Peusomyia*** Sæther, 1970

*Chaoborus pallidus* (Fabricius, 1794)

**sg. *Schadanophasma*** Dyar & Shannon, 1924

*Chaoborus nyblaei* (Zetterstedt, 1838)

***CRYOPHILA*** Edwards, 1930

*Cryophila lapponica* (Martini, 1928)

***MOCHLONYX*** Loew, 1844

*Mochlonyx fuliginosus* Felt, 1905

= *Mochlonyx martinii* Edwards, 1930

= *Mochlonyx velutinus* of authors

*Mochlonyx velutinus* (Ruthe, 1831)

= *Mochlonyx culiciformis* (De Geer, 1776) preocc.

**DIXIDAE** Schiner, 1868

***DIXA*** Meigen, 1818

*Dixa dilatata* Strobl, 1900

*Dixa nebulosa* Meigen, 1830

*Dixa puberula* Loew, 1849

*Dixa submaculata* Edwards, 1920

***DIXELLA*** Dyar & Shannon, 1924

*Dixella aestivalis* (Meigen, 1818)

*Dixella amphibia* (De Geer, 1776)

*Dixella autumnalis* (Meigen, 1838)

*Dixella borealis* (Martini, 1928)

*Dixella dyari* (Garret, 1924)

*Dixella filicornis* (Edwards, 1926)

*Dixella hyperborea* (Bergroth, 1889)

*Dixella laeta* (Loew, 1849)

*Dixella naevia* (Peus, 1934)

*Dixella nigra* (Staeger, 1840)

*Dixella obscura* (Loew, 1849)

*Dixella serotina* (Meigen, 1818)

**THAUMALEIDAE** Bezzi, 1913

***THAUMALEA*** Ruthe, 1831

*Thaumalea truncata* Edwards, 1929

infraorder Ptychopteromorpha Wood & Borkent, 1986

superfamily Ptychopteroidea Osten Sacken, 1862

**PTYCHOPTERIDAE** Osten Sacken, 1862

***PTYCHOPTERA*** Meigen, 1803

**sg. *Paraptychoptera*** Tonnoir, 1919

*Ptychoptera lacustris* Meigen, 1830

*Ptychoptera paludosa* Meigen, 1804

**sg. *Ptychoptera*** Meigen, 1803

*Ptychoptera albimana* (Fabricius, 1787)

*Ptychoptera contaminata* (Linnaeus, 1758)

*Ptychoptera hugoi* Tjeder, 1968

*Ptychoptera minuta* Tonnoir, 1919

*Ptychoptera scutellaris* Meigen, 1818

## Excluded species

*Dixa maculata* Meigen, 1818. There is one female specimen collected from Russia, Kantalaks (=Kandalaksha) that belongs to *Dixella borealis*.

## Notes

***Threticus tridactilis***: Ostrobotthnia borealis pars borealis, Rovaniemi, Nammalikkokivalo, 13.6.–11.7.2004, J. Salmela leg.; Regio kuusamoensis, Taivalkoski, Poikaloukusanoja, 1.6.–1.7.2006, J. Salmela leg.; Regio kuusamoensis, Taivalkoski, Syväoja 1.6.-1.7.2006, J. Salmela leg.

***Panimerus albifacies***: Regio aboensis, Turku, Pomponrahka-Isosuo mire conservation area, 1.8.–28.9.2011, J. Salmela & E. Nummela leg.

***Panimerus przhiboroi***: Ostrobotthnia borealis pars borealis, Tervola, Ruuttulammi conservation area, 4.6.–2.7.2012 J. Salmela leg. (New for European fauna)

***Psychoda albipennis* Zetterstedt** has been confirmed to occur in Finland by Bo-W. Svensson (female specimen from Tavastia australis, South Finland, Tampere, 2009, J. Salmela leg.).

***Dixella dyari***: Lapponia enontekiensis, Enontekiö, Pallas-Yllästunturi National Park, Lumikero E, 25.8.2010, J. Salmela leg.
